# A Modern Collared Cementless Femoral Stem for the Arthroplasty Treatment of Femoral Neck Fractures

**DOI:** 10.3390/jcm15062110

**Published:** 2026-03-10

**Authors:** Brandon H. Naylor, Mary Jane McConnell, Anita (Alex) Bradham, Natalie L. Gresham, Zachary M. Ricciardelli, Charlotte C. Baker, Brian E. Seng, Thomas L. Bradbury, Joseph M. Schwab

**Affiliations:** Total Joint Specialists, Cumming, GA 30041, USA

**Keywords:** hip fracture, total hip arthroplasty, hemiarthroplasty, osteoporosis, femoral neck fracture

## Abstract

**Background/Objectives**: For femoral neck fractures (FNFs) treated with hip arthroplasty, cemented femoral fixation is frequently recommended due to its association with reduced early perioperative fracture and reoperation rates. However, newer-generation collared, cementless triple-tapered (CCTT) stems may present benefits compared with conventional press-fit designs. This study sought to assess 30-day survivorship of a CCTT stem in patients undergoing hip arthroplasty for FNF via the direct anterior approach (DAA). **Methods**: We conducted a retrospective review of all patients who underwent hemiarthroplasty (HA) or total hip arthroplasty (THA) for displaced FNF from 2019 to 2023. All procedures were performed through the DAA using a hydroxyapatite-coated CCTT femoral stem. The primary outcome was 30-day stem survival. Secondary outcomes included reoperation, stem revision, complications, readmission, and radiographic outcomes including intraoperative and postoperative periprosthetic fracture and subsidence. **Results**: A total of 184 patients were included (mean age 76.1 ± 10.0; 70.1% women). THA was performed in 77.7% and HA in 22.3%. At 30 days, no stems required revision. The 30-day reoperation rate was 3.5% (5/184). There were four intraoperative fractures: 3 (1.6%) Vancouver AG and 1 (0.5%) calcar. Postoperatively within 3 months, seven fractures occurred: five (2.7%) Vancouver AG and two (1.1%) Vancouver B1. **Conclusions**: Use of a modern CCTT femoral stem for FNF demonstrated excellent early survivorship with low rates of intraoperative and postoperative periprosthetic fracture, comparable to published outcomes of cemented fixation. These findings suggest that this stem design may represent a safe and efficient alternative to cemented femoral fixation in FNF. Further studies are warranted to evaluate mid- and long-term outcomes.

## 1. Introduction

Femoral neck fractures (FNFs) are associated with high morbidity, mortality, and healthcare burden. Arthroplasty is the preferred management option for displaced FNFs, yet the optimal fixation method, cemented versus cementless, remains debated [1,2,3]. Cemented fixation reduces early periprosthetic fracture and revision rates, whereas cementless fixation avoids bone cement implantation syndrome and may reduce operative time and infection risk [2,4,5]. Recent advances in implant design have led to the development of modern collared, cementless, triple-tapered (CCTT) hydroxyapatite-coated femoral stems intended to improve initial fixation and mitigate early failure associated with traditional uncemented stems [6]. A recent study examining two-year outcomes with this stem design found a 50% lower revision rate, an 18% reduction in operating room time, and a 19% shorter hospital length of stay (LOS) compared to other designs [6]. The performance of this stem style in the setting of hip arthroplasty for FNF, however, has not been specifically evaluated. We therefore seek to investigate the outcomes of a modern CCTT stem for FNF. Specifically, we asked:

1. What is the 30-day stem survivorship?
2. What are the rates of reoperation, revision, readmission, and complications within 30 days?
3. What are the rates of intraoperative and 3-month postoperative periprosthetic fracture and early radiographic subsidence or loosening?

## 2. Materials and Methods

A retrospective Institutional Review Board-approved study was conducted using clinical data from consecutive patients who received a modern CCTT, hydroxyapatite-coated femoral stem (ACTIS, J&J MedTech, Warsaw, IN, USA) for acute displaced femoral neck fracture between January 2019 and June 2023. All procedures were performed by one of four fellowship-trained, high-volume arthroplasty surgeons within a single urban hospital system. Cemented fixation was not utilized in any case.

### 2.1. Inclusion and Exclusion Criteria

Patients were included if they were ≥18 years old, sustained a displaced FNF, and underwent primary THA or HA using the ACTIS femoral stem during the study period. Patients were excluded if they had <30 days of both clinical and radiographic follow-up, underwent arthroplasty for indications other than acute displaced FNF, or had incomplete records preventing outcome assessment.

### 2.2. Surgical Technique and Postoperative Protocol

All procedures were performed through the direct anterior approach (DAA). Femoral preparation followed ACTIS stem guidelines, with selective femoral neck reaming to optimize collar seating. When the neck cut was lower than the desired collar seating, no reaming was required. Broaching was done by hand or by using automated broaching devices, and intraoperative fluoroscopy confirmed stem alignment and collar contact. Prophylactic cables or wires were used based on surgeon preference. Postoperatively, patients were allowed unrestricted weight bearing as tolerated. Discharge disposition and subsequent level of care were determined based on individualized assessment by a licensed physical therapist (home with home therapy, outpatient therapy, acute inpatient rehabilitation, etc.).

### 2.3. Data Collection

Baseline characteristics included age, sex, mechanism of injury, American Society of Anesthesiologists (ASA) classification, and Dorr classification (Table 1). Perioperative variables collected included time from emergency department (ED) admission to operating room (OR), laterality, procedure type (THA or HA), estimated blood loss (EBL), operative time, stem size and offset, use of an automated stem impactor, intraoperative use of wires or cables, LOS, and follow-up duration (Table 2).

ED-to-OR time was calculated from ED admission to the surgical date. Operative time was defined as the time from skin incision to closure. LOS was measured from the surgical date to hospital discharge. Body mass index (BMI) was not consistently recorded during admission and was therefore excluded from analysis. Clinical and radiographic follow-up were measured from the surgical date to the most recent clinical visit or radiographic imaging. Patients with less than 30 days of both clinical and radiographic follow-up were excluded from the final analysis.

### 2.4. Radiographic Evaluation

All patients underwent intraoperative fluoroscopy, including anteroposterior (AP) pelvis and hip views. Standard postoperative radiographs were obtained at 2 weeks, 6 weeks, 3 months, and 1 year. Radiographs were assessed for Dorr classification, quantitative stem subsidence, loosening, and periprosthetic fracture.

Radiographic evaluation was performed independently by two reviewers who used a standardized assessment protocol; discrepancies were resolved by the senior author. The position of the stem collar relative to the calcar was measured on intraoperative fluoroscopy. Collar position was defined as any change in the distance from the base of the collar to the calcar and categorized as <1 mm (none), 1–3 mm, or >3 mm. Postoperative radiographs were evaluated for stem subsidence, and both intraoperative and postoperative images were reviewed for the presence of fractures. All fractures were classified using the Vancouver system. The most recent follow-up radiograph was used to evaluate for stem loosening, defined by radiolucent lines within femoral Gruen zones [7].

### 2.5. Clinical Evaluation

The primary endpoint was 30-day stem survivorship. Secondary outcomes included early clinical events (reoperation, revision, complications, and readmission) and radiographic outcomes assessed through 3 months. The 30-day interval for stem survivorship was selected due to the low expected rate of mechanical stem failure beyond this period [8] and the traditionally high rate of loss to follow-up in this population [9].

To minimize loss to follow-up, 15 patients were contacted: 8 voicemails (not included in the study), 6 positive responses (included if other inclusion criteria met), and 1 incorrect number (not included). Patients with dementia or altered mental status were not contacted. Details of revisions and prosthetic complications were documented. Risk factors for fracture, stem revision, and reoperation were assessed using perioperative variables including surgical procedure, automated stem impactor use, collar position, Dorr classification, age, and sex.

### 2.6. Data Analyses

Continuous variables were summarized as means with standard deviations or ranges, while categorical variables were presented as frequencies and percentages. Survivorship and clinical outcomes were assessed based on the most recent clinical and radiographic follow-up. Statistical analyses were conducted using Microsoft Excel (Microsoft Corporation, Redmond, WA, USA) and RStudio (2024.12.0, Posit Software PBC, Boston, MA, USA).

## 3. Results

### 3.1. Demographic and Surgical Data

A total of 184 hips in 184 patients were identified during the study period. The mean age was 76.1 (±10.0) years; 129 (70.1%) were women and 55 (29.9%) men. The primary mechanism of injury was ground-level fall (GLF) in 177 (96.2%) patients. ASA classifications included 36 (19.6%) ASA 2, 130 (70.7%) ASA 3, and 18 (9.8%) ASA 4. Dorr classification distribution was Type A = 28.1%, Type B = 62.4%, and Type C = 9.6% (Table 1).

The mean time from ED to OR was 1.5 (range: 0–14) days. A total of 94 (51.1%) left hips and 90 (48.9%) right hips were treated. THA was performed in 143 (77.7%) and HA in 41 (22.3%). Mean EBL was 280 ± 173 mL, and mean operative time was 55.7 min (41–86) for HA and 65.6 min (27–182) for THA. An automated broach impaction device (Kincise, J&J MedTech, Warsaw, IN, USA) was used in 92 (50.0%) cases. Prophylactic wires or cables were used in 14 (7.6%). The mean LOS was 4.4 (range: 0–20) days, and the mean clinical follow-up duration was 211 (range: 0–1409) days (Table 2).

### 3.2. 30-Day Outcomes

Patients were also evaluated at follow-up, and data on complications, reoperations, and readmissions were collected. Within 30 days, no patients (0.0%) required stem revision, 5 (2.7%) underwent reoperation, and 29 (15.8%) were readmitted (Table 3). Causes for 30-day reoperation included periprosthetic fracture in two (1.1%) patients, prosthetic joint infection in two (1.1%), and dislocation in two (1.1%) (one patient experienced both fracture and dislocation) (Table 4).

### 3.3. Fractures

There were four intraoperative fractures: three (1.6%) Vancouver AG and one (0.5%) calcar. Postoperatively, within 3 months, seven fractures occurred: five (2.7%) Vancouver AG and two (1.1%) Vancouver B1 (Table 5).

### 3.4. Subsidence and Loosening

Of the 184 patients, 177 (96.1%) were assessed radiographically for collar positioning. On fluoroscopic imaging, the femoral stem collar was flush with the calcar in 155 (87.6%) patients, positioned 1–3 mm above in 15 (8.5%), and >3 mm above in seven (4.0%) (Table 5). Stem subsidence of >3 mm was observed in two (1.1%) patients at the latest follow-up. No evidence of stem loosening was identified (Table 5).

### 3.5. Risk Factors

Univariate regression identified no risk factors for fracture within 3 months. No variables were significantly associated with 30-day stem revision. However, stem size 11 (OR 1.65, 95% CI 1.30–2.08; *p* < 0.001) and Dorr Type C femurs (OR 1.19, 95% CI 1.95–1.30; *p* < 0.001) were associated with increased risk of 30-day reoperation. No other demographic or implant-related factors, including arthroplasty type, collar position, use of an automated impaction device, age, or sex, were significant predictors of revision or reoperation within 30 days.

## 4. Discussion

We report on a series of patients undergoing THA or HA for FNF using a modern CCTT stem through the DAA. Multiple studies show acceptable results for the treatment of FNF using DAA [10,11,12]. However, those studies focused on the surgical approach learning curve or comparative analysis between surgical approaches. The focus of our series is to assess early stem stability and fracture risk in this vulnerable population using this popular, but relatively new stem design. In our series, 30-day stem survivorship was 100%. The intraoperative fracture rate was 2.2% (4/184), and the 3-month postoperative periprosthetic fracture rate was 3.8% (7/184). These results suggest that the CCTT stem provides stable early fixation and a low complication rate.

**Figure 1:**
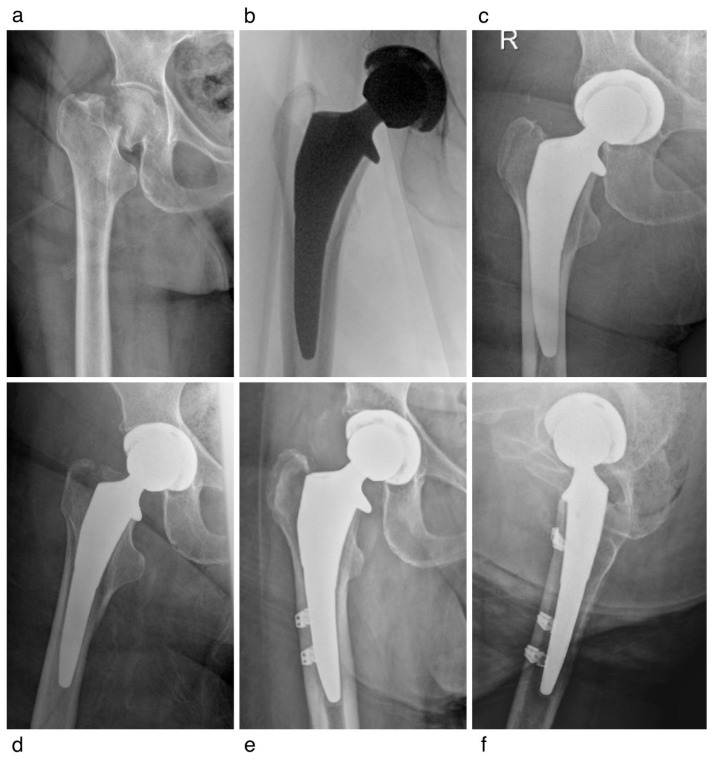
A 90-year-old woman initially presented with a femoral neck fracture. One week after primary fixation, a fall resulted in a periprosthetic Vancouver B1 fracture. The fracture was treated with three cerclage cables and demonstrated radiographic union at 3 months postoperatively. (**a**) Initial femoral neck fracture, anteroposterior (AP) view (**b**) Intraoperative AP view (**c**,**d**) Eight days postoperatively, showing Vancouver B1 periprosthetic fracture, AP and frog-leg lateral views (**e**,**f**) Three months postoperatively with healed fracture and three cerclage cables, AP and cross-table lateral view.

**Figure 2:**
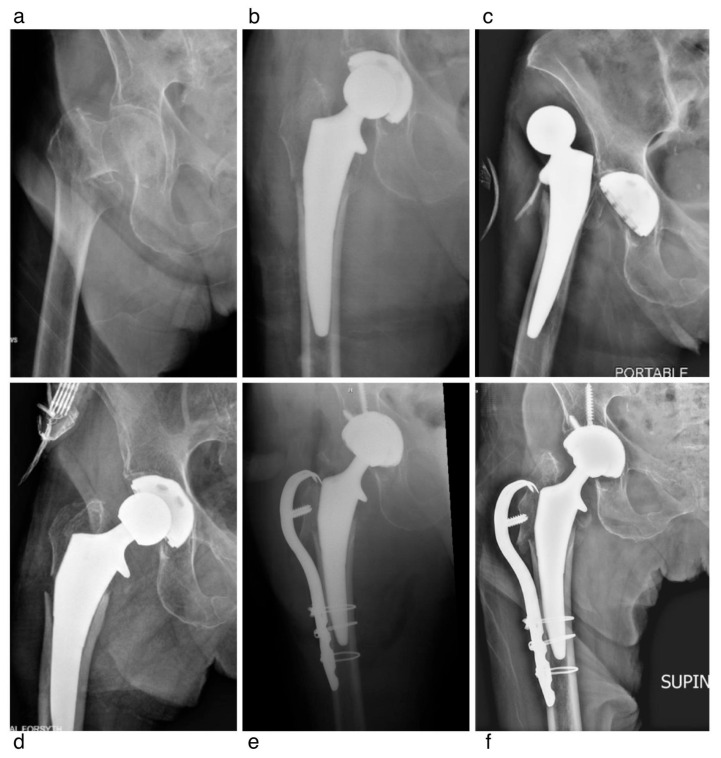
An 88 year-old woman with a femoral neck fracture. Intraoperatively, a Vancouver AG fracture occurred due to errant retractor placement; however, both the stem and fracture remained stable. Two weeks postoperatively, the patient sustained a fall resulting in a dislocation. Closed reduction was unsuccessful, and she was subsequently treated with open hip reduction, open reduction and internal fixation (ORIF) of the trochanteric fracture, and acetabular revision. (**a**) Femoral neck fracture, AP view; (**b**) intraoperative greater trochanter fracture, AP view; (**c**) two-week postoperative dislocation, AP view; (**d**) successful open hip reduction, AP view; (**e**,**f**) postoperative ORIF of trochanteric fracture and acetabular revision, AP views.

Two patients (Patients One and Two) represented our only reoperations for periprosthetic fracture within 30 days. Patient One, a 90-year-old woman, sustained a minimally displaced Vancouver B1 periprosthetic fracture 1 week after primary fixation for FNF. The fracture occurred following a fall and was successfully managed with three cerclage cables, demonstrating a healed fracture on the radiograph by 3 months postoperatively (Figure 1). Patient Two, an 88-year-old woman, sustained an intraoperative Vancouver AG fracture due to errant retractor placement; both the fracture and the femoral stem were stable initially. However, a fall at 2 weeks postoperatively resulted in a dislocation. After an unsuccessful closed reduction, she underwent open hip reduction, open reduction and internal fixation (ORIF) of the greater trochanteric fracture, and acetabular revision (Figure 2).

This study represents the largest reported series evaluating a modern CCTT stem for FNF. Prior reports of cementless fixation for FNF have shown revision rates ranging from 4.3% [13] to 52.2% [14] primarily due to periprosthetic fracture. Our results compare favorably, supporting that a CCTT stem may reduce implant failure and the need for early revision.

Recent AAOS guidelines recommend cemented fixation in older adults, based largely on a review of studies of earlier collarless, blade-style stems [15]. Veldman et al. (2017) [5] reported higher complication rates with cementless stems, including periprosthetic fracture, loosening, and dislocation. Taylor et al. (2012) [16] also favor cemented fixation, while Figved et al. (2009) [15] and Moerman et al. (2017) [17] found similar functional outcomes between fixation types. Santini et al. (2005) [18] and Inngul et al. (2013) [19] likewise showed comparable mortality and function between groups.

Overall, most literature suggests cemented stems may reduce early implant-related complications [20,21,22,23,24,25,26,27], yet functional outcomes are generally equivalent [17,18]. Fixation choice should therefore depend on patient factors, surgeon experience, and institutional preference [15,19,28]. It is also worth noting that cemented fixation carries the risk of bone cement implantation syndrome [29].

### Limitations

This study is limited by its retrospective design and the absence of a cemented or older cementless comparison cohort, which restricts the ability to make direct comparisons or definitive conclusions regarding equivalence or superiority. Comparisons were therefore limited to previously published outcomes from other studies. Additionally, follow-up duration was relatively short, which limits assessment of longer-term complications, including implant survivorship. There was also a high rate of loss to long-term follow-up, which is typical in the elderly femoral neck fracture population. All procedures were performed by high-volume arthroplasty surgeons, which may limit generalizability to other practice settings. Selection bias was minimized by including all sequential FNFs treated with cementless fixation during the study period.

## 5. Conclusions

Use of a modern CCTT stem for FNF demonstrates excellent early survivorship, with no stem revisions within 30 days and a low rate of intraoperative and postoperative periprosthetic fracture. When used for both THA and HA through the DAA, the implant provides stable fixation and favorable short-term safety. These results suggest that contemporary collared cementless designs may serve as an effective alternative to cemented fixation for FNF. Further mid- to long-term studies are warranted to evaluate implant longevity and functional outcomes.

## Figures and Tables

**Table 1:** Patient Demographics.

	Patients (N = 184)
Age (years, mean ± SD)	76.1 ± 10.0
Gender	
Female	129 (70.1%)
Male	55 (29.9%)
Mechanism of Injury	
Ground-level fall	177 (96.2%)
Trauma	4 (2.2%)
Other	3 (1.6%)
ASA Classification	
2	36 (19.6%)
3	130 (70.7%)
4	18 (9.8%)
Dorr Classification	
A	50 (28.1%)
B	111 (62.4%)
C	17 (9.6%)

SD, standard deviation; ASA classification, American Society of Anesthesiologists classification.

**Table 2:** Patient surgical data.

	Patients (N = 184)
Mean Time from ED to OR (days) (range)	1.5 (0–14)
Laterality	
L	94 (51.1%)
R	90 (48.9%)
Procedure	
Hemiarthroplasty	41 (22.3%)
Total Hip Arthroplasty	143 (77.7%)
Mean EBL (mL, mean ± SD)	280 ± 173
Mean Operative time (minutes)	
Hemiarthroplasty	55.7 (41–86)
Total Hip Arthroplasty	65.6 (27–182)
Stem Size	
2	0 (0.0%)
3	11 (6.0%)
4	19 (10.4%)
5	34 (18.6%)
6	37 (20.2%)
7	37 (20.2%)
8	20(10.9%)
9	11 (6.0%)
10	11 (6.0%)
11	2 (1.1%)
12	1 (0.5%)
Stem Offset	
Standard	125 (67.9%)
High	59 (32.1%)
Automated impaction device used	92 (50.0%)
Wires or Cables Used	14 (7.6%)
Mean Length of Stay (days) (range)	5.2 (0–20)
Mean Follow-up (days) (range)	211 (0–1409)

SD, standard deviation; ED, emergency department; OR, operating room; EBL, estimated blood loss.

**Table 3:** Complications, 30-day readmissions, and reoperations.

	Patients (N = 184)
Stem Revision (30 days)	0 (0.0%)
Readmission (30 days)	29 (15.8%)
Reoperation (30 days)	5 (2.7%)
Fracture Requiring Reoperation (30 days)	2 (1.1%)
PJI Requiring Reoperation (30 days)	2 (1.1%)
Dislocation Requiring Reoperation (30 days)	2 * (1.1%)
Medical Complication (90 days)	48 (26.1%)
Death (During Follow-up Period)	17 (9.2%)
PJI, Periprosthetic Joint Infection	

* one patient experienced a fracture and a dislocation requiring reoperation within 30 days.

**Table 4:** 30-Day Complications.

Sex	Age	Complication	Time Point	Treatment Provided	Outcome
F	90	(Figure 1) Vancouver B1 Fracture	1 Week	ORIF	Fracture Healed Uneventfully
F	88	(Figure 2) Vancouver AG Fracture and Dislocation	2 Weeks	Open Hip Reduction, Revision THA Acetabulum, ORIF	No Further Dislocations and Fracture Healed Uneventfully
F	71	Dislocation	3 Weeks	Open Reduction	No Further Dislocation
M	61	PJI	4 Weeks	I&D with Head Exchange	No Signs of Continued Infection
M	68	PJI	4 Weeks	I&D with Poly Exchange	No Signs of Continued Infection

THA, total hip arthroplasty; ORIF, open reduction and internal fixation; I&D, incision and drainage.

**Table 5:** Intraoperative fractures, postoperative fractures, collar position, subsidence, and loosening.

	Patients (N = 184)
Intraoperative Fractures	
None	180 (97.8%)
Vancouver AG	3 (1.6%)
Calcar	1 (0.5%)
Fractures within 3 Months	
None	177 (96.2%)
Vancouver AG	5 (2.7%)
Vancouver B1	2 (1.1%)
Collar Position Immediate Post-op (N = 177)	
Flush	155 (87.6%)
1–3 mm proud	15 (8.5%)
>3 mm proud	7 (4.0%)
Subsidence (>3 mm)	2 (1.1%)
Loosening	0 (0.0%)

## Data Availability

The data presented in this study are available on request from the corresponding author due to privacy restrictions.

## Abbreviations

The following abbreviations are used in this manuscript:

FNF	Femoral neck fracture
CCTT	Collared, cementless triple-taper
LOS	Length of stay
DAA	Direct anterior approach
THA	Total hip arthroplasty
HA	Hemiarthroplasty
ASA	American Society of Anesthesiologists
ED	Emergency department
OR	Operating room
EBL	Estimated blood loss
BMI	Body mass index
AP	Anteroposterior
ORIF	Open reduction and internal fixation
SD	Standard deviation
PJI	Periprosthetic joint infection
I&D	Incision and drainage
